# Cohesin loss and MLL-AF9 are not synthetic lethal in murine hematopoietic stem and progenitor cells

**DOI:** 10.21203/rs.3.rs-3894962/v1

**Published:** 2024-01-29

**Authors:** Alison Meyer, Cary Stelloh, Nan Zhu, Sridhar Rao

## Abstract

Objective As cohesin mutations are rarely found in MLL-rearranged acute myeloid leukemias, we investigated the potential synthetic lethality between cohesin mutations and MLL-AF9 using murine hematopoietic stem and progenitor cells. Results Contrary to our hypothesis, a complete loss of *Stag2* or haploinsufficiency of *Smc3* were well tolerated in MLL-AF9-expressing hematopoietic stem and progenitor cells. Minimal effect of cohesin subunit loss on the *in vitro* self-renewal of MLL-AF9-expressing cells was observed. Despite the differing mutational landscapes of cohesin-mutated and MLL fusion AMLs, previous studies showed that cohesin and MLL fusion mutations similarly drive abnormal self-renewal through *HOXA* gene upregulation. The utilization of a similar mechanism suggests that little selective pressure exists for the acquisition of cohesin mutations in AMLs expressing MLL fusions, explaining their lack of co-occurrence. Our results emphasize the importance of using genetic models to test suspected synthetic lethality and suggest that a lack of co-occurrence may instead point to a common mechanism of action between two mutations.

### Materials and Methods

*Mice and HSPC isolation*

The *Stag2*$^{fl/fl}$ and *Smc3*$^{fl/+}$ animals were generated as described (1, 2). Mice were genotyped and excision assays were performed as described in Viny et al. 2015 and Viny et al. 2020 (1, 2). Whole bone marrow was isolated from the tibias, femurs, and pelvic bones of 8-12 week old mice and, following red cell lysis, HSPCs were isolated using the mouse lineage depletion kit (Miltenyi Biotec, 130-090-858).

*Plasmids, retroviral production, and infection*

Retroviral plasmids for empty vector (MSCV puro, #68469) and tamoxifen-inducible Cre (ERT-Cre, #22776) were purchased from Addgene. pMSCV MLL-AF9-IRES-GFP was a gift from Scott Armstrong. 293T cells were transfected with MLL-AF9, MSCV puro, or ERT-Cre alongside the packaging vector pCL-Eco (Addgene #12371). Viral media was harvested 48 and 72 hours post-infection. Infections were done by spinoculation on retronectin-coated plates as described in Milanovich et al. 2015 (3). Cells were sorted 48 hours following MLL-AF9 infection for GFP+. Cells were then spinoculated again with MSCV puro or ERT-Cre virus and selected in 1 mg/mL puromycin. 24 hours post-infection, cells were treated with 2 nM 4-hydroxytamoxifen (Tocris) to induce Cre expression.

*Self-renewal assays*

1,000 HSPCs were plated in triplicate in 35mm dishes in methylcellulose (MethoCult M3434, Stemcell Technologies). Cells were grown for 7 days after which colonies were enumerated, resuspended, and replated (1,000 cells) for serial passaging.

*qPCR*

Total RNA was isolated from 1 million HSPCs using Trizol (ThermoFisher Scientific, 15596018). cDNA was synthesized using the iScript kit (BioRad, 170-8891). qPCR was performed in triplicate using the SYBR green mastermix (BioRad, 172-5124) on a QuantStudio6 machine using the following primers:

Mouse *Stag2* exon 6-7F: CTGCAAAGGAGTCGTCACAG; Mouse *Stag2* exon 6-7R: ACTGCCGTACTAACACACCA

Mouse *Smc3*F: CAGAGCAGCGATTGGCTTTG; Mouse *Smc3*R: AGATGTGTTGGCCTCATCCA

Mouse b-actin F: GATCTGGCACCACACCTTCTACAATG; Mouse b-actin R: ACCGGTTGTCCGAATTGTCA

Relative mRNA expression was calculated using the DDCt method using b-actin normalization.

*Western blotting*

Western blotting was performed as described in Meyer et al. 2022 (4). The following antibodies were used: anti-b-Actin (Millipore-Sigma, A5441, 1:2000), anti-Smc3 (Bethyl, A300060, 1:1000), and anti-Stag2 (Santa Cruz, sc-81852, 1:1000).

*Flow cytometry*

Measurements of GFP+ were collected on a BDFACS Celesta machine and analysis was performed with FlowJo v10.8.1.

## Introduction

Translocations involving the MLL1 gene (*KMT2A*) are associated with the development of acute myeloid leukemia (AML) and result in the expression of many different MLL fusion proteins, of which MLL-AF9, MLL-AF4, and MLL-ENL are the most common (5). MLL fusions are robust oncogenes and can transform both hematopoietic stem and progenitor cells (HSPCs) and more committed progenitors (5). MLL fusions are found in approximately 5–10% of patients with AML and drive HSPC self-renewal and leukemic transformation (5, 6). Despite the large degree of genetic complexity found in AML (7), AMLs with MLL fusions have a small number of co-operating mutations and display a clear preference for the acquisition of *RAS* family mutations, including *KRAS*, *NRAS*, *PTPN11*, and *BRAF* (5, 6, 8–10).

Approximately 10% of patients with AML have a mutation in one of four cohesin complex members (*STAG2*, *SMC1A*, *SMC3*, or *RAD21*) (7, 11–15). Cohesin mutations are mutually exclusive and result in haploinsufficiency and increased HSPC self-renewal (7, 13–16). Despite their similar rates of overall occurrence, MLL fusions and cohesin mutations are rarely observed together. The rate of cohesin mutations in all MLL fusion AMLs is approximately 7% (cBioportal OHSU Cancer Cell 2022 and TCGA NEJM 2013 datasets and (6, 8, 17)). The rate of co-occurrence is particularly low in AMLs harboring MLL-AF9 (cBioportal, 2.7% and (8)). Unlike MLL-AF9, cohesin mutations do not often co-occur with *RAS* family mutations and instead co-occur most often with *NPM1* mutations (7, 12, 13, 18). The difference in co-occurrence between cohesin and MLL-AF9 or *NPM1* is surprising considering both MLL-AF9 and *NPM1*-driven leukemias are known to be reliant on increased *HOXA* expression (19–22). Interestingly, *NPM1* mutations are not observed within MLL-AF9 leukemia (10). Due to the low incidence of MLL-AF9 and cohesin mutations in AML and their differing mutational patterns, we hypothesized that cohesin mutations would be detrimental to hematopoietic stem and progenitor cells harboring MLL-AF9. To this end, we expressed MLL-AF9 in HSPCs isolated from mice bearing floxed alleles of the cohesin subunits *Stag2* (2) or *Smc3* (1) and performed self-renewal assays in excised vs. unexcised cells. In contrast to our hypothesis, we found that both a complete loss of *Stag2* as well as haploinsufficiency of *Smc3* were well tolerated by cells expressing MLL-AF9. While a significant increase in self-renewal was observed with MLL-AF9 expression alone, the addition of a cohesin mutation did not reduce or largely enhance self-renewal in MLL-AF9 expressing cells.

## Results

### Stag2 and MLL-AF9 mutations are not mutually exclusive

The gene encoding S*tag2* is X-linked. In contrast to the other cohesin subunits (Smc3, Rad21, and Smc1a), a complete loss of Stag2 is tolerated due to partial compensation by Stag1 (2, 23–26). To test for mutual exclusivity between Stag2 and MLL-AF9 mutations, we isolated lineage depleted bone marrow cells (HSPCs) from a female *Stag2*$^{fl/fl}$ mouse and retrovirally transduced these cells with *MLL-AF9-IRES-GFP*. Following sorting for GFP+, cells were infected with either empty vector or tamoxifen-inducible Cre. Following 4-OHT treatment to excise both copies of *Stag2*, self-renewal assays were performed *in vitro* in methylcellulose. Cells receiving empty vector were also treated with 4-OHT. Wild type (WT) HSPC colony numbers (Colony Forming Units; CFUs) decreased with serial passaging, as expected (Fig. 1A). Consistent with previous publications, *Stag2*$^{-/-}$ HSPCs showed enhanced self-renewal over WT starting at passage 2 (Fig. 1A)(2, 14, 27). *Stag2*$^{fl/fl}$ HSPCs (unexcised) expressing MLL-AF9 also showed enhanced self-renewal over both WT and *Stag2*$^{-/-}$ cells (Fig. 1A). Excision of *Stag2* in the presence of MLL-AF9, however, did not result in loss of self-renewal capacity (Fig. 1A *MLL-AF9;Stag2*$^{fl/fl}$ vs. *MLL-AF9;Stag2*$^{-/-}$). In contrast, at quaternary passage, an increase in self-renewal in *MLL-AF9*;*Stag2*$^{-/-}$ cells over *MLL-AF9* alone was observed (Fig. 1A). Collectively, our data indicate that the loss of *Stag2* had a small but statistically significant increase in abnormal self-renewal initiated by MLL-AF9 and that *Stag2* loss is supported in MLL-AF9-expressing cells.

It is possible that the parental line contained a small number of cells with incompletely excised *Stag2* that were selected for with serial passaging. To ensure that full excision of both *Stag2* alleles was maintained across all passages, we examined *Stag2* excision by PCR, where the unexcised, floxed allele runs at 554 bp and the excised allele runs at 294 bp (**Supplemental Fig. 1A**) (2). To confirm the sensitivity of our assay, we mixed unexcised *Stag2*$^{fl/fl}$ and excised *Stag2*$^{-/-}$ DNA controls at varying molar ratios and performed the excision PCR. A slight 554 pb band was detectable with a 10% spike-in of unexcised DNA, which was readily detectable at 20% (**Supplementary Fig. 1A**). No unexcised band was detectable in the parental line (*MLL-AF9;Stag2*$^{-/-}$) or at any passage (**Supplementary Fig. 1A**). As exon 7 is excised upon Cre exposure (2), we also performed qPCR using primers spanning the exon 6–7 junction. We readily detected transcript in WT cells, but no transcript was detectable in *Stag2*$^{-/-}$ control cells or in our *MLL-AF9;Stag2*$^{-/-}$ cells throughout serial passaging, confirming that no read-through was occurring and supporting complete excision (**Supplementary Fig. 1B**). Western blotting confirmed a lack of *Stag2* expression throughout serial passaging (Fig. 1B). Full length blots are presented in Supplementary Fig. 1C. Finally, as *Stag2*$^{-/-}$ cells also show enhanced self-renewal, it is possible that MLL-AF9 was lost with serial passaging. However, cells remained GFP + throughout the assay as determined by flow cytometry, indicating retention of the retroviral cassette (**Supplementary Fig. 1D-F**). We conclude that *Stag2* was fully excised, and that this excision was maintained throughout serial passaging. Further, no synthetic lethality was observed between *MLL-AF9* and *Stag2* in murine HPSCs.

### Smc3 haploinsufficiency and MLL-AF9 are not mutually exclusive

In contrast to *Stag2*, a complete loss of *Smc3* is lethal, and AMLs harboring *Smc3* mutations are functionally haploinsufficient (1, 13). To determine if *Smc3* haploinsufficiency is also tolerated in MLL-AF9-expressing HSPCs, we isolated HSPCs from an *Smc3*$^{fl/+}$ mouse and infected these cells with *MLL-AF9-IRES-GFP* retrovirus. Following sorting for GFP + cells, cells were transduced with empty vector or tamoxifen-inducible Cre virus. Following 4-OHT treatment of both empty vector and Cre-transduced cells, self-renewal was analyzed by serial replating assays. Similar to *Stag2*$^{-/-}$, *Smc3*$^{-/+}$ HSPCs showed enhanced self-renewal over WT HSPCs (Fig. 2A). *MLL-AF9;Smc3*$^{fl/+}$ cells (unexcised) also showed enhanced self-renewal, as was observed in our Stag2 assays (Fig. 2A). Excision of one copy of *Smc3* in the presence of MLL-AF9 did not result in a loss of self-renewal capacity (Fig. 2A). To check for Smc3 excision, we performed a PCR assay where the excised Smc3 allele is 349 bp, the unexcised, floxed allele is 313 bp, and the WT allele is 287 bp. *MLL-AF9;Smc3*$^{-/+}$ cells maintained excised and heterozygous throughout serial passaging (**Supplementary Fig. 2A**). Due to the heterozygosity of the cells, we were unable to perform a similar spike-in experiment to check excision sensitivity as was done in *Stag2*$^{-/-}$ cells. We performed qPCR for *Smc3* and found a consistent 60–70% loss of Smc3 mRNA throughout serial passaging, consistent with excision of one Smc3 allele (**Supplementary Fig. 2B**). Consistent with our previous results, western blotting also showed a loss of Smc3 protein compared to WT cells throughout serial passaging (Fig. 2B,C)(4). While Smc3 protein levels slightly increased over serial passaging, the level never rose above that observed for our independent *Smc3*$^{-/+}$ control (Fig. 2B,C). Smc3 protein expression in our *MLL-AF9;Smc3*$^{-/+}$ cells remained similar to that observed in a known *Smc3*$^{-/+}$ control (Fig. 2B,C). As with the Stag2 experiment, cells remained GFP + throughout serial passaging as determined by flow cytometry (**Supplementary Fig. 2C,D)**. Compare to GFP- control **Supplementary Fig. 1D**). We conclude that a loss of a single allele of *Smc3* is compatible with *MLL-AF9* in murine HSPCs.

## Discussion

In AML, MLL fusions do not co-occur readily with cohesin mutations, but instead co-occur with Ras family mutations (5, 6, 8–10). In contrast, cohesin mutations tend to co-occur with *NPM1* mutations but not with Ras family mutations (7, 12, 13, 18). Further, *NPM1* mutations are not found in MLL-fusion AMLs (10). Based on these different mutational landscapes, we hypothesized that the addition of cohesin haploinsufficiency to MLL-AF9-expressing HSPCs would be detrimental. Contrary to this hypothesis, we found that both a complete loss of *Stag2* and *Smc3* haploinsufficiency were well tolerated by MLL-AF9-expressing cells. While *Stag2* loss increased the self-renewal capacity of MLL-AF9-expressing cells at later passages (Fig. 1A), minimal effects on self-renewal were observed upon excision of one allele of *Smc3* (Fig. 2A). Thus, despite their predicted synthetic lethality and differing co-mutational spectra in AML, MLL-AF9 and cohesin mutations are permissible for HSPC growth and self-renewal.

Interestingly, both cohesin haploinsufficient and MLL-AF9 HSPCs and AMLs have elevated expression of *HOXA* cluster genes, particularly *HOXA7* and *9*, which are critical for enhanced self-renewal (5, 28, 29). *HOXA9* is also important for AML transformation and maintenance (28, 30, 31). Knockdown of *HOXA9* reduces proliferation in primary human MLL-fusion AMLs through cell cycle arrest, increased differentiation, and increased apoptosis (28). Over-expression of *HOXA9* in hematopoietic stem and progenitor cells results in stem cell expansion and leukemic development, suggesting that both MLL-AF9 and cohesin mutations co-opt key regulators of *HOXA* gene expression (28).

In support of this, both MLL-AF9 and cohesin-mutated cells influence gene expression through interactions with the DOT1L and PRC2 histone methyltransferase complexes, respectively (5, 29, 32). MLL-AF9 recruits the DOT1L methyltransferase to target genes such as HOXA9 (5). DOT1L deposits the activating mark H3K79me2, which drives gene expression. In hematopoietic cells, *HOXA7/9* are repressed by the PRC2 complex, which deposits the repressing mark H3K27me3 at promoter regions. The cohesin subunits Rad21 and Smc3 have been shown to interact with components of the PRC2 complex, with cohesin loss resulting in decreased PRC2 recruitment to murine *Hoxa7/9* promoters (29). This allows for Dot1L recruitment and enhanced gene activation. Interestingly, DOT1L is critical for both the initiation and maintenance of MLL-AF9 AML, and small molecule inhibitors of DOT1L are particularly effective against mouse and human MLL-rearranged leukemias in vitro and in xenograft models (31–34). Furthermore, the abnormal self-renewal observed in murine HSPCs depleted of cohesin were restored by Dot1l inhibition (35). Collectively, these data suggest that DOT1L inhibitors may be particularly effective against MLL-rearranged or cohesin-mutated AMLs.

Because MLL-AF9 is a strong driver of *HOXA* gene expression and self-renewal and, unlike Rad21-depletion, up-regulates the HOXA9 co-factor MEIS1 (28, 29), there is likely little selective pressure for the acquisition of cohesin mutations in MLL-AF9-expressing AMLs, potentially explaining why they are not commonly observed together. Interestingly, *NPM1* mutations are more commonly observed with cohesin mutations and, like MLL-AF9, *NPM1* also drives *HoxA* expression. This suggests that additional mechanisms beyond HOXA-induced self-renewal may contribute to *NPM1*-driven leukemias, and such mechanisms are supported by cohesin haploinsufficiency. We have shown one such mechanism is through Rac-regulated growth and apoptosis (4). While it is important to consider that differences may exist in *in vitro* vs. *in vivo* assays as well as between mice and humans, our experiments suggest that the absence of co-occurrence, while often interpreted as synthetic lethality, may instead reflect significant overlap in the molecular mechanisms driving the observed disease biology. Intriguingly, both cohesin mutant and MLL-AF9 AMLs are sensitive to DOT1L inhibitors. Thus, a lack of co-occurrence may point to a common, targetable mechanism behind two initially dissimilar appearing leukemias, underlining the importance of genetically testing mutual exclusivity.

## Limitations

Assays were performed on HSPCs isolated from a single *Stag2*$^{fl/fl}$ or *Smc3*$^{fl/+}$ mouse. Each *Stag2*$^{fl/fl}$ and *Smc3*$^{fl/+}$ parental cell line, once infected with MLL-AF9 and treated with 4-OHT, was followed over serial re-passaging, and while some assays were repeated (serial replating), others were not (qPCR, genotyping, western blot, or GFP + assays). Thus, differences may exist between individual mice or with long-term culture, which would not be revealed by our assays. However, the consistency between our results in *Stag2*$^{-/-}$ and *Smc3*$^{-/+}$ cells and between passages does not support this. In addition, Fig. 2B and C show that Smc3 protein levels did slightly increase between passage 1 and 4, suggesting a selection for cells with higher Smc3 expression may occur in the presence of MLL-AF9. However, the level of Smc3 expression did not rise above that of an *Smc3*$^{-/+}$ control. Further experiments would need to be performed to determine if higher Smc3 expression is selected for in the presence of MLL-AF9. Additionally, as mentioned in our discussion section, all of our assays are performed *in vitro*, and different results may be observed *in vivo.* Lastly, our experiments focus primarily on leukemogenesis. Thus, loss of Stag2 or Smc3 may contribute to aspects of leukemia maintenance that were not addressed in this manuscript.

## Abbreviations

AML
Acute Myeloid Leukemia
HSPC
Hematopoietic Stem and Progenitor Cells
4OHT
4-Hydroxytamoxifen
WT
Wild type

CFUs
Colony Forming Units

## Declarations

#### Competing interests:

The authors have no financial or non-financial competing interests to report. Ethics approval: All animal studies were approved by the Medical College of Wisconsin Institutional Animal Care and Use Committee. Availability of data and materials: All materials and raw data will be made available upon reasonable request. Funding: This work was funded by: NCI R01 CA204231 to S. Rao. AEM is supported by a generous gift from Ms. Nan Gardetto. Author contributions: AEM performed experiments, analyzed data, and wrote the manuscript, CS performed mouse husbandry and genotyping experiments and edited the manuscript, NZ provided MLL-AF9 constructs, advice, and edited the manuscript. SR supervised experiments and edited the manuscript. Acknowledgements: The authors would like to acknowledge Benedetta Bonacci for performing cell-sorting.

## Author Contribution

AEM performed experiments, analyzed data, and wrote the manuscript, CS performed mouse husbandry and genotyping experiments and edited the manuscript, NZ provided MLL-AF9 constructs, advice, and edited the manuscript. SR supervised experiments and edited the manuscript.

## Figures

Figure 1

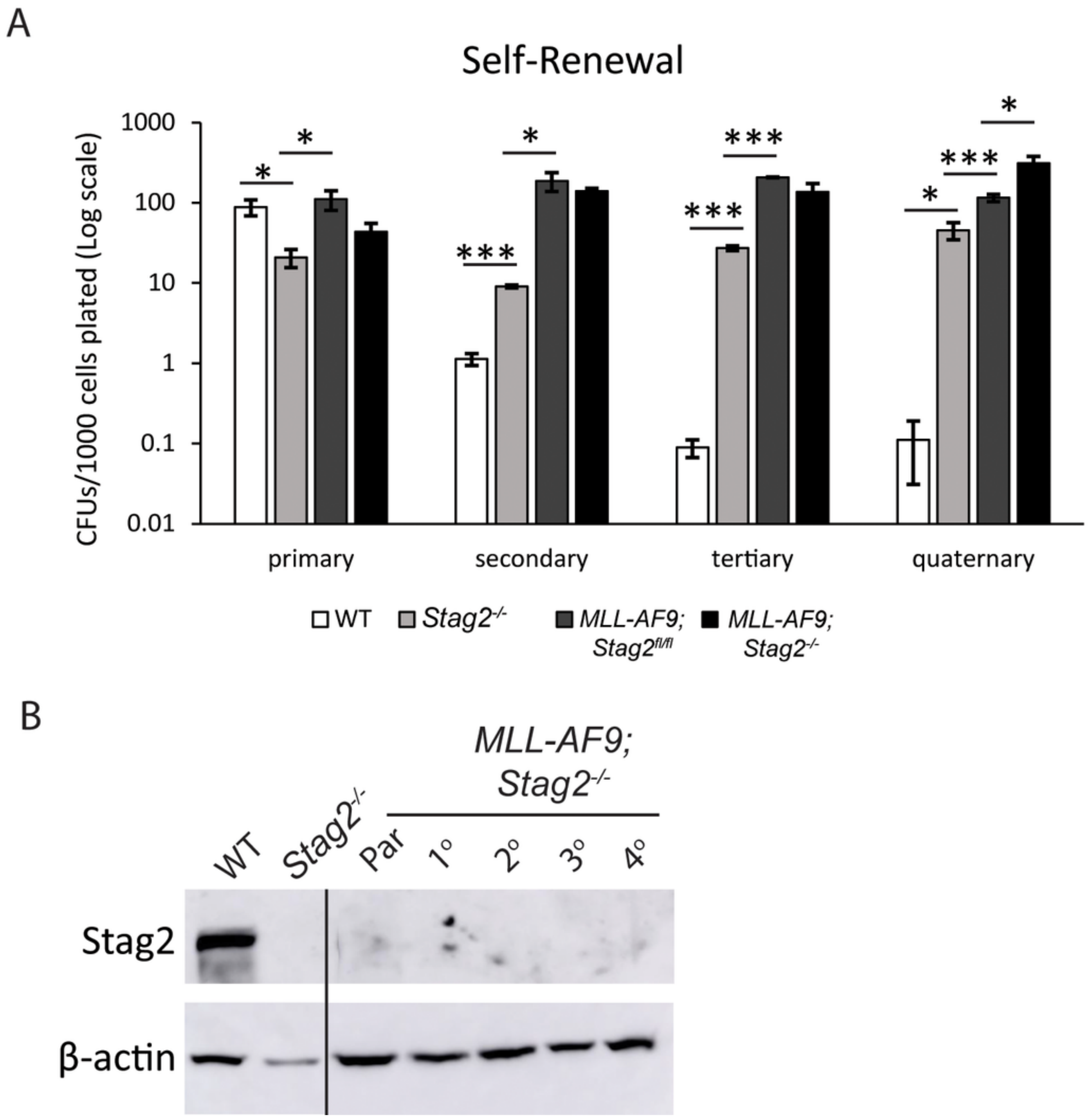

**Figure 1**

A) 1000 HSPCs of the indicated genotypes were plated in methylcellulose and colonies were allowed to grow for 7 days between passages. Colonies were counted (CFUs) and 1000 cells were re-plated with each passage. Colony numbers in *MLL-AF9;Stag2*$^{fl/fl}$ (empty vector transduced) were compared with those of *MLL-AF9;Stag2*$^{-/-}$ (tamoxifen-Cre transduced) and WT and *Stag2*$^{-/-}$ controls. Both *MLL-AF9;Stag2*$^{fl/fl}$ and *MLL-AF9;Stag2*$^{-/-}$ cells were treated with tamoxifen. * $p<0.05$, *** $p<0.001$ Student's ttest. B) Western blot showing Stag2 expression (top blot) in WT cells and a lack of Stag2 expression in the *Stag2*$^{-/-}$ control and in *MLL-AF9;Stag2*$^{-/-}$ cells throughout serial passaging. “Par” refers to the parental line prior to plating in methylcellulose. Expression was compared to b-actin (bottom blot). The black line indicates where intervening lanes were removed for clarity. The uncropped blot is available in Supplementary Figure 1C.

Figure 2

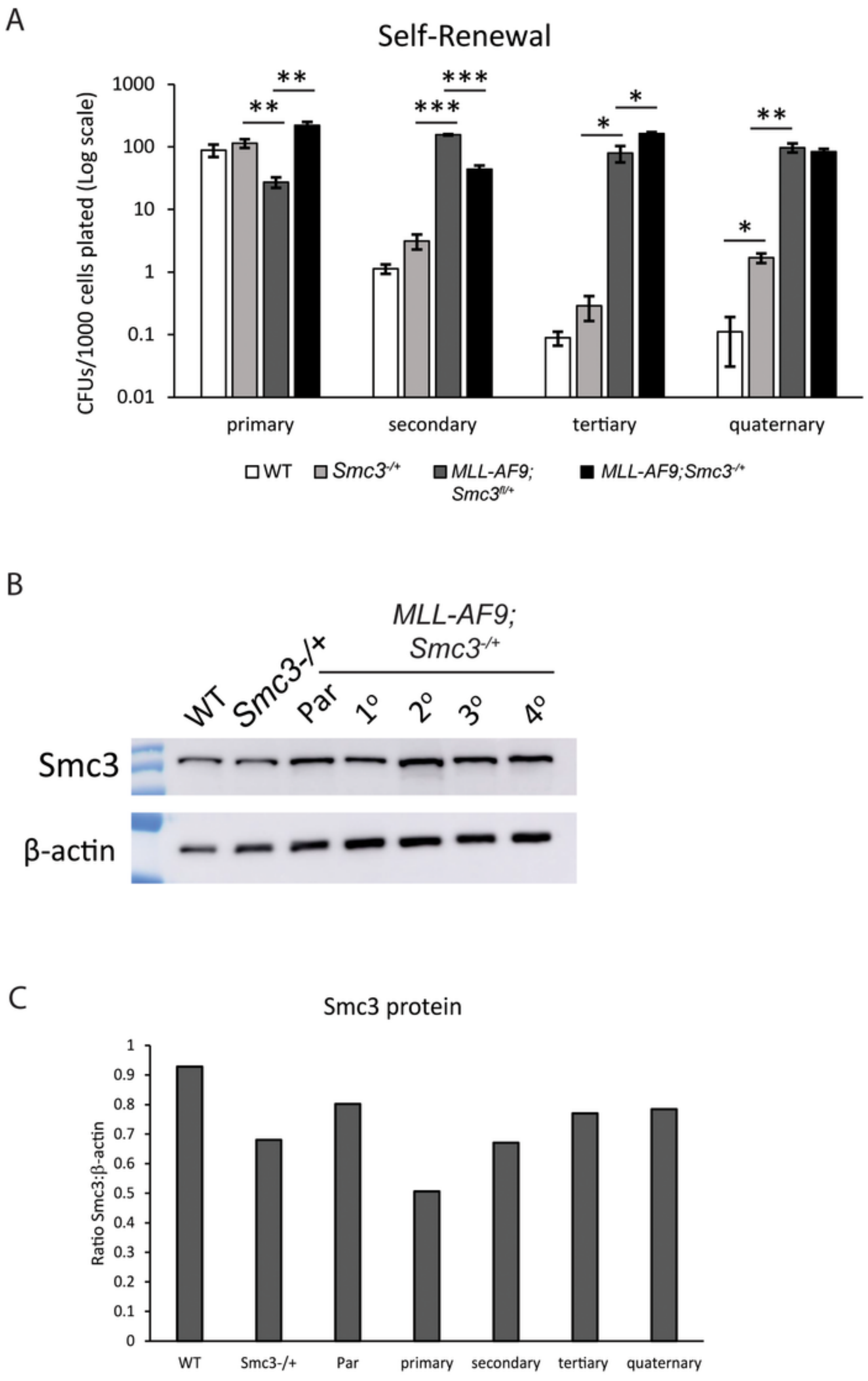

**Figure 2**

A) 1000 HSPCs of the indicated genotypes were plated in methylcellulose and colonies were allowed to grow for 7 days between passages. Colonies were counted (CFUs) and 1000 cells were re-plated with each passage. Colony numbers in *MLL-AF9;Smc3*$^{fl/+}$ (empty vector transduced) were compared with those of *MLL-AF9;Smc3*$^{-/+}$ (tamoxifen-Cre transduced) and WT and *Smc3*$^{-/+}$ controls. Both *MLL-AF9;Smc3*$^{fl/+}$ and *MLL-AF9;Smc3*$^{-/+}$ cells were treated with tamoxifen. * p<0.05, *p<0.01, *** p<0.001 Student's t-test. B) Western blot showing Smc3 expression (top blot) in WT and *Smc3*$^{-/+}$ controls and in *MLL-AF9;Smc3*$^{-/+}$ cells throughout serial passaging. "Par" refers to the parental line prior to plating in methylcellulose. Expression is compared to b-actin (bottom blot). C) Bands from B were quantified by densitometry using ImageJ and the ratios of Smc3:b-actin were plotted. The graph shows an approximate 20-30% loss of Smc3 expression in *Smc3*$^{-/+}$ and *MLL-AF9;Smc3*$^{-/+}$ cells throughout serial passaging. "Par" refers to the parental line prior to plating in methylcellulose.

## Supplementary Files

This is a list of supplementary files associated with this preprint. Click to download.

- SupplementaryFigure1.tif
- SupplementaryFigure2.tif
- Uncroppedblots.tif
- SupplementaryFigure1uncroppedgel.tif
- Supplementaryfigure2uncroppedgel.tif
